# The landscape of circulating tumor HPV DNA and TTMV-HPVDNA for surveillance of HPV-oropharyngeal carcinoma: systematic review and meta-analysis

**DOI:** 10.1186/s13046-024-03137-1

**Published:** 2024-08-03

**Authors:** Flaminia Campo, Oreste Iocca, Francesca Paolini, Valentina Manciocco, Silvia Moretto, Armando De Virgilio, Claudio Moretti, Antonello Vidiri, Aldo Venuti, Paolo Bossi, Giovanni Blandino, Raul Pellini

**Affiliations:** 1 Department of Otolaryngology-Head and Neck Surgery, IRCCS Regina Elena National Cancer Institute, Istituti Fisioterapici Ospitalieri (IFO), Via Elio Chianesi 53, Rome, 00144 Italy; 2 Division of Maxillofacial Surgery, Surgical Science Department, University of Torino, Torino, Italy; 3 HPV- Unit, UOSD Tumor Immunology and Immunotherapy IRCCS Regina Elena National Cancer Institute, Istituti Fisioterapici Ospitalieri (IFO), Rome, Italy; 4 Deparment of Biochemical Sciences A. Rossi Fanelli, Sapienza University of Rome, Rome, Italy; 5 Department of Biomedical Sciences, Humanitas University, Milan, Italy; 6 Otorhinolaryngology Unit, IRCCS Humanitas Research Hospital, Milan, Italy; 7 Department of Radiology and Diagnostic Imaging, IRCCS Regina Elena National Cancer Institute, Istituti Fisioterapici Ospitalieri (IFO), Rome, Italy; 8 IRCCS Humanitas Research Hospital, via Manzoni 56, Rozzano, Milan, 20089 Italy; 9 Department of Biomedical Sciences, Humanitas University, Via Rita Levi Montalcini 4, Pieve Emanuele, Milan, 20072 Italy; 10 Translational Oncology Research Unit, Department of Research, Diagnosis and Innovative Technologies, IRCCS Regina Elena National Cancer Institute, Rome, Italy

**Keywords:** Oropharyngeal squamous cell carcinoma, Liquid biopsy, Circulating tumour HPVDNA, HPV, TTMV-HPVDNA, Follow up

## Abstract

**Background:**

Human papilloma virus (HPV) related cancers of the oropharynx are rapidly increasing in incidence and may soon represent the majority of all head and neck cancers. Improved monitoring and surveillance methods are thus an urgent need in public health.

**Main text:**

The goal is to highlight the current potential and limitations of liquid biopsy through a meta analytic study on ctHPVDNA and TTMV-HPVDNA. It was performed a Literature search on articles published until December 2023 using three different databases: MEDLINE, Embase, and Cochrane Library. Studies that evaluated post-treatment ctHPVDNA and TTMV-HPVDNA in patients with HPV + OPSCC, studies reporting complete data on the diagnostic accuracy in recurrence, or in which the number of true positives, false positives, true negatives, and false negatives was extractable, and methods of detection of viral DNA clearly defined.

The meta-analysis was conducted following the Meta-analysis Of Observational Studies in Epidemiology (MOOSE) reporting guidelines.

The aim of this meta-analysis was to evaluate the sensitivity, specificity, and accuracy of ctHPVDNA and TTMV by ddPCR to define its efficacy in clinical setting for the follow up of HPV-OPSCC.

**Conclusion:**

The 12 studies included in the meta-analysis provided a total of 1311 patients for the analysis (398 valuated with ctHPVDNA and 913 with TTMV-HPVDNA). Pooled sensitivity and specificity were 86% (95% CI: 78%-91%) and 96% (95% CI: 91%-99%), respectively; negative and positive likelihood ratios were 0.072 (95% CI: 0.057–0.093) and 24.7 (95% CI: 6.5–93.2), respectively; pooled DOR was 371.66 (95% CI: 179.1–918). The area under the curve (AUC) was 0.81 (95% CI, 0.67–0.91).

Liquid biopsy for the identification of cell free DNA might identify earlier recurrence in HPV + OPSCC patients. At the present time, liquid biopsy protocol needs to be standardized and liquid biopsy cannot yet be used in clinical setting. In the future, a multidimensional integrated approach which links multiple clinical, radiological, and laboratory data will contribute to obtain the best follow-up strategies for the follow-up of HPV-OPSCC.

**Supplementary Information:**

The online version contains supplementary material available at 10.1186/s13046-024-03137-1.

## Introduction

Incidence of oropharyngeal squamous cell carcinoma (OPSCC) is rising exponentially in high-income countries [1], despite the decreased exposure to classic risk factors associated with the development of head neck cancers, namely cigarette smoking and alcohol consumption. This epidemiological trend can be attributed to an epidemic spread of high-risk oncogenic Human Papillomavirus (HPV) infection, a well-known risk factor for the development of oropharyngeal squamous cells carcinoma [2]. Of the over 200 genotypes currently known, 13 are associated with the development of neoplastic pathology in humans. Among these, the most well-known and studied is the HPV-16, which is responsible for almost 90% of these cases [3]. The increase in incidence of HPV + OPSCC is so exponential that the number of men affected by HPV-OPSCC has surpassed the number of women affected by HPV-related cervical carcinoma, making OPSCC the most commonly HPV-related cancer in industrialized countries [4].

Despite the ongoing evolution of treatment modalities with the introduction of robotic surgery, the diagnostic workup has not evolved for several years [5].

Regarding follow-up, the current National Comprehensive Cancer Network guidelines indicate the execution of imaging at baseline after treatment and clinical assessment at regular intervals for a minimum of five years. Positron emission tomography (PET) at 3 months after completion of chemoradiation is considered standard of care [6]. However, over time, several critical issues have emerged regarding this surveillance modality. For instance, it has been highlighted that the use of PET scans in post-radio chemotherapy treatment is characterized by a high number of false positives [7–9]. PET-CTs have a poor positive predictive value of 30% on 12 week surveillance for HPV-OPSCC [10]. A recent meta-analysis highlighted that PET-CT results were equivocal for 22.5% (95% CI, 12.5–36.9) and equivocal/positive for 34.2% of patients (95% CI, 25.1–44.5) [11].

Even when combining this method with Magnetic Resonance Imaging (MRI), distinguishing between disease persistence and normal post-treatment metabolic response remains complicated [9, 12, 13]. Furthermore, the use of cyto/histological typing through fine needle aspiration in these cases is characterized by a failure rate of approximately 30% [14, 15].

The use of multiple visits leads to increased costs for the national healthcare system and the development of anxiety and depression for the patients [16].

On the other hand, an early and precise disease diagnosis coupled with a timely treatment is likely associated with better overall survival [17].

Due to this gray area in the diagnostic workup, the search for new biomarkers has risen over the years, and an increasing number of studies are investigating the utility of liquid biopsy at diagnosis and during follow up. In detail, circulating tumor HPVDNA (ctHPVDNA) and circulating tumor tissue–modified viral HPV DNA (TTMV-HPVDNA) are emerging as promising biomarkers to improve clinical decision- making in the care of OPSCC patients.

Although several academic groups have developed research- grade circulating tumor HPV DNA (ctHPVDNA) assays, the first commercial ctHPVDNA assay, based on detection of circulating tu- mor tissue–modified viral HPV DNA (TTMV-HPV DNA), became available in the USA in 2020 and allowed for wide- spread clinical practice to this technology [18].

Previous meta-analyses demonstrated that digital drop PCR (ddPCR) for ctHPVDNA has good accuracy, sensitivity and specificity for first diagnosis of HPV-related OPSCC [19].

However, a recent narrative review on TTMV-HPVDNA and ctHPVDNA development for early detection of cancer recurrence highlights existing knowledge gaps and suggests research that should be prioritized to understand the association between biomarker-based surveillance and patient outcomes [18].

In this setting we elaborate a systematic review and meta-analytic study on ctHPVDNA and TTMV-HPVDNA, to highlight the current potential and limitations of liquid biopsy.

Thus, the aim of this meta-analysis is to evaluate the sensitivity, specificity, and accuracy of ctHPV DNA and TTMV by ddPCR to define its efficacy in the clinical setting for the follow up of HPV-OPSCC.

## Materials and methods

Systematic review and meta-analysis were conducted following the Meta-analysis Of Observational Studies in Epidemiology (MOOSE).

### Study eligibility criteria

The inclusion criteria were as follows: 1) studies that evaluated post-treatment ctHPVDNA and TTMV-HPVDNA in patients with HPV + OPSCC, 2) studies reporting complete data on the diagnostic accuracy in recurrence, or in which the number of true positives, false positives, true negatives, and false negatives was extractable, 3) methods of detection of viral DNA clearly defined.

The exclusion criteria were: 1) incomplete data on the patients’ follow up; 2) non-original studies (i.e., reviews). Peer-reviewed publications in English were included, with no restrictions to the publication year.

### Search strategy

Authors conducted a literature search on articles published until December 2023 using three different databases: MEDLINE, Embase, and Cochrane Library searching for studies examining the diagnostic performance of ctHPVDNA and TTMV during follow up in patients with HPV-related OPSCC (Supporting Table 1).

**Table 1:** Main features of the included studies

**Author (year)**	**Patiens**	**Male (n, %)**	**Subsite**	**Age (mean)**	**cT**	**cN**	**cM**	**Stage (AJCC VIII ed.)**	**Treatment**	**Deintesification treatment (n, %)**
Akashi, K. (2022) [20]	25	22 (88%)	/	66	T1:9 T2:11 T4:5	N0:2 N1:19 N2:4	M0:25	I:19 II:1 III:3 IV: 2	Surgery:10 RT:8 Induction CT-surgery:1 Induction CT-RT:6	/
Berger, B., M. (2022) [21]	1076	943 (87.6%)	1076: OPSCC	63	/	/	M0:1076	/	/	/
Chera, B., S. (2020) [22]	115	101 (87.8%)	/	/	/	/	M0:115	I:86 II:18 III:11	CRT:93 RT:22	97 (88%)
Ferrier, S., T. (2023) [23]	60	49 (81.7%)	36: tonsil 24: BOT	66	/	/	/	/	Induction CT-surgery:42 CRT:14 Others:4	42 (70%)
Haring, C., T. (2021) [24]	27	/	27:OPSCC	/	T1:14 T2:13	/	M0:27	/	Surgery:27	/
Jakobsen, K. K. (2023) [25]	72	60 (83.3%)	45: tonsil 23: BOT 3: others	62	T1:24 T2:29 T3:9 T4:10	N0:13 N1:44 N2:15	M0:72	I:45 II:16 III:11	RT:60 Surgery: 11 Surgery-RT:1	/
O'Boyle, C. J. (2022) [26]	49	43 (87.7%)	27: tonsil 20: BOT 1: CUP 2: overlapping		T0:1 T1:23 T2:22 T3:2 T4:1	N0:4 N1:38 N2:3 N3:4	M0:49	/	CRT:15 Surgery:10 Surgery-RT:16 Surgery-CRT:8	/
Tatsumi et al. (2024) [27]	23	19 (82.6%)	/	67	/	/	M0:23	I:17 II:4 III:2	RT:23	/
Warlow, S. J. (2022) [28]	104	73 (70%)	58: tonsil 37: BOT 9: others	61	T0:5 T1:31 T2:27 T3:9 T4:32	N0:14 N1:15 N2:73 N3:2	M0:104	/	RT:13 CRT:75 Surgery:9 Palliative:4 Induction CT-CRT: 3	
Tanaka, H. (2021) [29]	35	29 (82.8%)	26: OPSCC 2: CUP 4: Hipopharynx 4: Nose 1: Larynx	68	T0:2 T1:4 T2:16 T3:6 T4:7	N0:4 N1:5 N2:26	M:0	I:2 II:6 III:27	RT:23 CRT:8 Induction CT-RT:4	/
Ferrandino, R., M. (2023) [30]	290	237 (81.7%)	290:OPSCC	63	T0:14 T1:107 T2:121 T3:27 T4:21	N0:39 N1:196 N2:51	/	/	Surgery:71 Surgery-RT:109 RT:7 CRT:54 Induction CT-CRT:49	/
Hanna, G. J. (2023) [31]	543	466 (86%)	267: Tonsil223: BOT23: Overlapping30: CUP	61	T0:25T1:184T2:214T3:82T4:37	N0:58N1:367N2:106N3:11	M0:543	/	Surgery:121CRT:227Surgery-RT:84Surgery-CRT:81Other:30	/

The articles were surveyed applying the selection criteria on the title and abstract (phase 1) and then on the full text of those deemed appropriate after the first analysis (phase 2). In addition, a manual search was conducted for references from the selected studies. Duplicate abstracts were carefully removed.

### Data extraction

A standardized electronic data collection form was used independently by two reviewers (FC, CM) to extract the data from each of the included studies such as the first author’s name, year of publication, study design, country, number of patients, cancer site, HPV status of cancer, number of pretreatment blood tests, HPV status in blood and method for the detection of viral DNA.

The extracted outcomes about the diagnostic accuracy of ctHPVDNA as a detection test for disease progression in patients affected by HPV-positive HNSCC were the number of true positives, false positives, true negatives, and false negatives.

### Statistical analysis

A diagnostic random effects meta-analysis was carried out using the DerSimonian-Laird method. The pooled sensitivity and specificity, the diagnostic odds ratio (DOR), positive and negative likelihood ratios were calculated. Results were reported with a 95% confidence interval (CI) for all the analyses. A correction factor of 0.5 for “0” events was applied. A subgroup meta-analysis was also executed dividing the studies in two groups according to the diagnostic method used, ctHPV DNA or TTMV HPV DNA. All the analyses were performed using R software for statistical computing (R 2.10.1; “meta” and “mada” package).

### Risk of bias

The Quality Assessment of Diagnostic Accuracy Studies second edition (QUADAS-2) was applied to calculate the potential risk of bias and quality of included studies. The seven items of QUADAS-2 checklist were scored in all included articles. The risk of bias was rated high (H), low (L), or unclear (U) according to the QUADAS-2.

## Results

### Study selection

The preliminary search, according to the scheme defined, led to the identification of 438 articles. After the removal of duplicates, 189 articles were detected. All the 189 publication were screened in title and abstract and 49 papers were revised in full text. No other relevant articles were identified from the reference screening. Twelve articles, published between 2019 and 2023, fully met the inclusion criteria for the statistical analysis [20, 21, 23, 25, 26, 29, 31–33].

### Probes for HPV cDNA detection by ddPCR

All studies used droplet digital PCR (ddPCR) and all studies extracted circulating tumour DNA from plasma. Clinical and demographical data is reported in Table 1. Primers/probes used by the studies included were different, as showed in Table 2.

**Table 2:** Main features of liquid biopsy test

**Author (year)**	**Study design**	**Patiens**	**Primers/probes**	**Siero/plasma**	**Methods**	**Liquid biopsy at diagnosis (n, %)**	**Positive liquid biopsy at diagnosis (n, %)**	**Test timing**
Akashi, K. (2022) [20]	prospective	25	E6—E7 probes HPV-16—18	plasma	ddPCR	25 (100%)	14 (56%)	/
Berger, B., M. (2022) [21]	prospective	1076	TTMV-HPVDNA probes HPV 16, 18, 31, 33, 35	plasma	ddPCR	/	/	At least one test during f/u
Chera, B., S. (2020) [22]	prospective	115	E6 e E7 probe HPV-16, E7 probes HPV 18, 31, 33, 35	plasma	ddPCR	86 (74.8%)	86 (100%)	1 – 3 months/1st year 2 – 6 months/2nd year 4 – 8 months/3rd year
Ferrier, S., T. (2023) [23]	prospective	60	E7 probes HPV 16–18-33–31-45	plasma	ddPCR	35 (58.3%)	32 (91.4%)	/
Haring, C., T. (2021) [24]	prospective	27	HPV16 ctDNA assay	plasma	ddPCR	27 (100%)	14 (51.8%)	1 month post treatment – every 3 months
Jakobsen, K. K. (2023) [25]	prospective	72	E6-E7 probes HPV 16–18-31–33-35–45-51–58	plasma	ddPCR	72 (100%)	70 (97.2%)	2 weeks—6 – 9 – 12 – 18 – 30 months
O'Boyle, C. J. (2022) [26]	prospective	49	E7 probes HPV 16–18-31–33-45	plasma	ddPCR	49 (100%)	48 (98%)	Surgery: 1 – 7 – 30 POD – 3 – 12 months CRT: 1/week during treatment – 3 – 12 months
Tatsumi et al. (2024) [27]	prospective	22	E6—E7 probe HPV-16	plasma	ddPCR	23 (100%)	22 (95.6%)	Every 10 Gy during treatment – 2- 4 months/after treatment
Warlow, S. J. (2022) [28]	prospective	104	E7 probes HPV 16—18—31—33—35	plasma	ddPCR	48 (46.2%)	48(100%)	/
Tanaka, H. (2021) [29]	prospective	35	E6—E7 probe HPV-16	plasma	ddPCR	30 (85.7%)	29 (96.7%)	3 months after RT and when recurrence become evident
Ferrandino, R., M. (2023) [30]	retrospective	290	TTMV-HPVDNA probes HPV 16, 18, 31, 33, 35	plasma	ddPCR	51 (17.6%)	51(100%)	At least one test during f/u
Hanna, G. J. (2023) [31]	retrospective	543	TTMV-HPVDNA probes 16, 18, 31, 33, 35	plasma	ddPCR	112 (21%)	96 (86%)	At least one test during f/u

### ctHPVDNA meta analysis for diagnostic accuracy in OPSCC

**Fig. 1:**
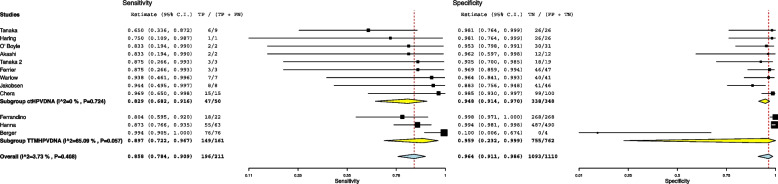
Diagnostic accuracy of ctHPVDNA and TTMV-HPVDNA displayed by forest plots estimating (**A**) sensitivity, **B** specify during follow up in patients with HPV + OPSCC (confidence interval (CI) in brackets)

**Fig. 2:**
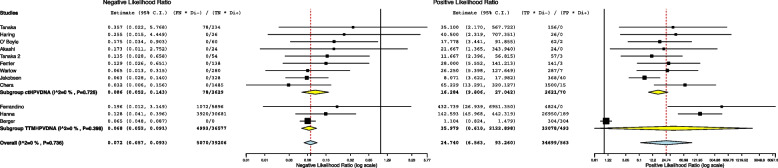
Diagnostic accuracy of ctHPVDNA and TTMV-HPVDNA displayed by forest plots estimating positive likelihood ratio (PLR), and negative likelihood ratio (NLR) during follow up in patients with HPV + OPSCC (confidence interval (CI) in brackets)

**Fig. 3:**
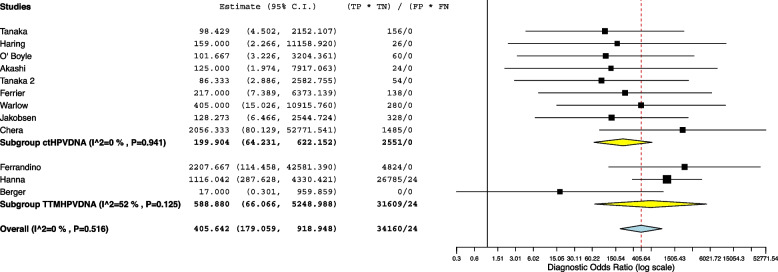
Diagnostic accuracy of ctHPVDNA and TTMV-HPVDNA displayed by forest plots estimating diagnostic odds ratio (DOR) during follow up in patients with HPV + OPSCC (confidence interval (CI) in brackets)

**Fig. 4:**
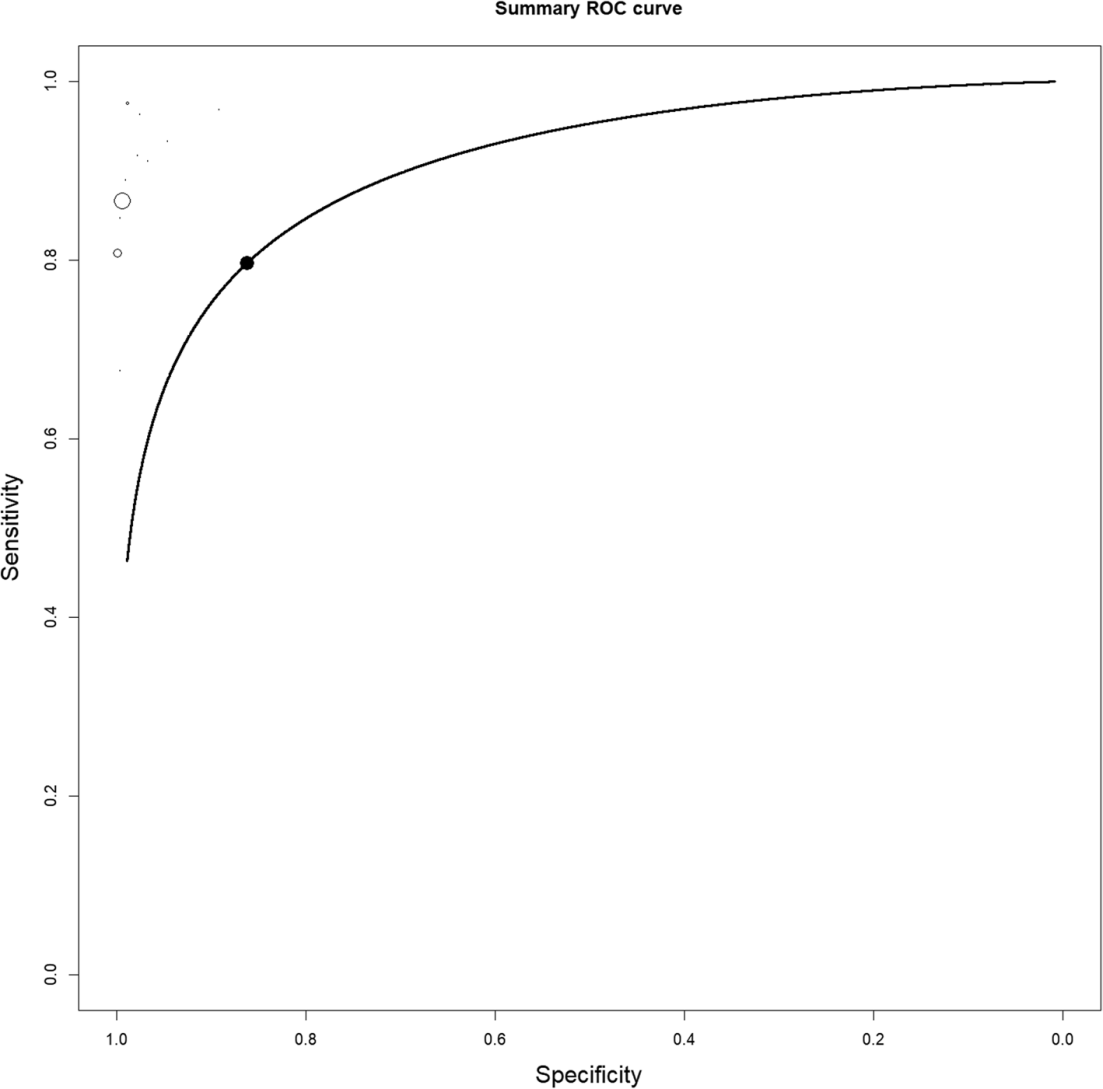
HSROC curve: the area under the curve (AUC) was 0.81 (95% CI 0.67–0.93)

The 12 studies included in the meta-analysis provided a total of 1311 patients for the analysis. The meta-analytic study estimated diagnostic performance of ctHPVDNA and TTMV during follow up as follows: pooled sensitivity and specificity of 86% (95% CI: 78%-91%) and 96% (95% CI: 91%-99%) (Fig. 1), respectively; negative and positive likelihood ratios of 0.072 (95% CI: 0.057–0.093) and 24.7 (95% CI: 6.5–93.2) (Fig. 2), respectively; pooled DOR of 371.66 (95% CI: 179.1–918.1) (Fig. 3). HSROC curve is presented as a supplementary figure ( Fig. 4). The area under the curve (AUC) was 0.81 (95% CI, 0.67–0.91).

The subgroup meta-analysis did not show a statistically significant difference among the ctHPVDNA and the TTMV-DNA subgroup both regarding sensitivity and specificity. The ctHPVDNA subgroup sensitivity was 82.9% (95% CI 68.2–91.6) while the TTMV-DNA was 89.7% (95% CI 72.2–96.7), *p* > 0.05. The ctHPVDNA subgroup specificity was 94.8% (95% CI 91.4–97.0) while the TTMV-DNA was 96.4% (95% CI 91.1–98.6), *p* > 0.05.

### Qualitative assessment

Quality assessment based on the QUADAS-2 is shown in Table 2s, and the overall risk of bias was rated low. Included studies fulfilled the items “patient selection”, “index test”, “reference standard”, and “flow and timing” of the risk of the bias section and all three items of the applicability concerns section (“patient selection”, “index test”, and “reference standard”).

## Discussion

Former meta-analyses on the diagnostic accuracy of liquid biopsy with the research of cell free DNA revealed that this technology is improving diagnostic protocol for several cancer including gastric cancer [34], lung cancer [35] and Head and Neck cancer [19, 36]. To the best our knowledge, this is the first meta-analysis exploring the accuracy of ctHPVDNA and TTMV-HPVDNA by ddPCR in patients with HPV + OPSCC during follow up. This meta-analysis analyzed outcomes from 1311 HPV + OPSCC patients: 398 valuated with ctHPVDNA and 913 with TTMV-HPVDNA. The results of the present meta-analysis indicate that the ctHPVDNA and TTMV-HPVDNA tests have the potential to be good diagnostic tools during follow-up. The goodness of a diagnostic test is based on multiple outcomes. First of all, the sensitivity and specificity values, followed by the likelihood ratio, the diagnostic odds ratio, and the ROC curves values. The pooled sensitivity and specificity of 86% (95% CI: 78%-91%) and 96% (95% CI 91%-99%) indicate that the test might be useful in clinical practice. Also, the positive and negative likelihood ratios (LR), which are a measure of diagnostic accuracy, gave satisfactorily results [37]. Good diagnostic tests have LR +  > 10 and have LR- < 0,1 [38]. Our meta-analysis shows LR + values of 24.7 (95% CI: 6.5–93.2) and LR- of 0.072 (95% CI: 0.057–0.093). These values correspond to a good diagnostic test. The diagnostic odds ratio (DOR) gives a rough estimate of diagnostic accuracy [39]. A value above 200 is generally accepted as those of a good diagnostic test, from our analysis a DOR of 371.66 was calculated. Regarding the meta-analysis by subgroup, no statistically significant difference between ctHPV DNA and TTMV-HPVDNA was evidenced. The ctHPVDNA subgroup sensitivity was 82.9% (95% CI 68.2–91.6) while the TTMV-HPVDNA was 89.7% (95% CI 72.2–96.7), *p* > 0.05. The ctHPVDNA subgroup specificity was 94.8% (95% CI 91.4–97.0) while the TTMV-DNA was 96.4% (95% CI 91.1–98.6), *p* > 0.05. HPV-related cancers of the oropharynx are rapidly increasing in incidence and may soon represent the majority of all head and neck cancers. Improved monitoring and surveillance methods are thus an urgent need in public health. Currently, the follow-up protocol for OPSCC patients is limited to imaging evaluation and the low diagnostic value and accuracy of such surveillance method may expose patients to unnecessary surgery [40]. Consequently, patients with HPV- associated OPSCC are prone to experience unnecessary diagnostic or therapeutic procedures, such as neck dissection. The rate of unnecessary neck dissection in case of clinical partial nodal response is high, and almost 60% of neck dissection specimens did not include cancer tissue [41]. Recently, researchers and clinicians have begun to evaluate the clinical utility of ctHPVDNA and TTMV-HPVDNA in biological fluids for the diagnosis and monitoring of patients with HPV-positive cancers. Tumor progression is associated with the expression of oncogenic viral DNA and proteins. Interestingly, EBV circulating DNA load is currently considered a new biomarker that reflects prognosis and change in response to nasopharyngeal cancer treatment [42]. It is thus reasonable that ctHPVDNA could have the same diagnostic/prognostic impact/efficacy. ctHPVDNA and TTMV-HPVDNA may have a role in diagnosis to confirm the correlation of the tumor with HPV [19], and during follow up to identify recurrence, as is evident from the current analysis. Furthermore, the kinetics of ctHPVDNA allows identifying the molecular residue disease [26] and in this setting liquid biopsy is used to select patients in de-escalation protocols [43]. It must be pointed out that meta-analysis has some limitations. First, the low number of studies somewhat limit the generalizability of results. Moreover, some heterogeneity between the included studies must be taken in consideration. ctHPVDNA assays are home-made and study design and primers/probes are different. In detail several studies analyze only HPV 16 while others have the possibility of identifying different strains of HPV. On the other the number of patients evaluated with TTMV-HPVDNA is more than double that with ctHPVDNA (913 vs 398). Furthermore, the assay used is always the same and this makes the methodology easier to evaluate. However, an important limitation is that two studies based on TTMV-HPVDNA are retrospective. Finally, a further limitation is the heterogeneity of the timing chosen to perform the test during follow-up, for this reason it is desirable that the liquid biopsy protocol is standardized.

In conclusion, this meta-analysis demonstrated that liquid biopsy have good accuracy, sensitivity and specificity for the diagnosis of relapse in patient with HPV + OPSCC. In the future, a multidimensional integrated approach which links multiple clinical, radiological, and laboratory data will contribute to obtain the best follow-up strategies for the follow-up of HPV-OPSCC. Currently caution is advised, liquid biopsy protocol needs to be standardized and liquid biopsy cannot yet be used in clinical setting. It is necessary to improved sensitivity before widespread adoption. In the next years, studies on larger and detailed patients’ cohorts and continued improvements in assay methodology and technology could allow the implementation of ctHPVDNA in routine clinical use.

### Supplementary Information

Supplementary Material 1: Table 1s. Algorithm for each database (MEDLINE, EMBASE, and Cochrane Library databases).

Supplementary Material 2: Table 2s. Assessment of methodological quality according to the Quality Assessment of Diagnostic Accuracy.

## Data Availability

All data generated or analyzed in this work are included in this article and/or its figures. Further enquiries can be directed to the corresponding author.

## Abbreviations

OPSCC: Oropharyngeal squamous cell carcinoma
HPV: Human Papillomavirus
PET: Positron emission tomography
MRI: Magnetic Resonance Imaging
ctHPVDNA: Circulating tumor HPVDNA
TTMV-HPVDNA: Circulating tumor tissue–modified viral HPV DNA
ddPCR: Digital drop PCR

## References

[1] Johnson DE, Burtness B, Leemans CR, Lui VWY, Bauman JE, Grandis JR. Head and neck squamous cell carcinoma. Nat Rev Dis Primers. 2020;6(1):92. 33243986 10.1038/s41572-020-00224-3 PMC7944998

[2] Mehanna H, Beech T, Nicholson T, El-Hariry I, McConkey C, Paleri V, et al. Prevalence of human papillomavirus in oropharyngeal and nonoropharyngeal head and neck cancer–systematic review and meta-analysis of trends by time and region. Head Neck. 2013;35(5):747–55. 22267298 10.1002/hed.22015

[3] Dona MG, Pichi B, Rollo F, Gheit T, Laquintana V, Covello R, et al. Mucosal and cutaneous human papillomaviruses in head and neck squamous cell papillomas. Head Neck. 2017;39(2):254–9. 27618734 10.1002/hed.24575

[4] Lechner M, Jones OS, Breeze CE, Gilson R. Gender-neutral HPV vaccination in the UK, rising male oropharyngeal cancer rates, and lack of HPV awareness. Lancet Infect Dis. 2019;19(2):131–2. 30722999 10.1016/S1473-3099(18)30802-8

[5] Campo F, Iocca O, De Virgilio A, Mazzola F, Mercante G, Pichi B, et al. Treatment of oropharyngeal squamous cell carcinoma: is swallowing quality better after TORS or RT? Radiother Oncol. 2023;183:109547. 36813176 10.1016/j.radonc.2023.109547

[6] Mehanna H, Wong WL, McConkey CC, Rahman JK, Robinson M, Hartley AG, et al. PET-CT surveillance versus neck dissection in advanced head and neck cancer. N Engl J Med. 2016;374(15):1444–54. 27007578 10.1056/NEJMoa1514493

[7] Corpman DW, Masroor F, Carpenter DM, Nayak S, Gurushanthaiah D, Wang KH. Posttreatment surveillance PET/CT for HPV-associated oropharyngeal cancer. Head Neck. 2019;41(2):456–62. 30549345 10.1002/hed.25425

[8] Rulach R, Zhou S, Hendry F, Stobo D, James A, Dempsey MF, et al. 12 week PET-CT has low positive predictive value for nodal residual disease in human papillomavirus-positive oropharyngeal cancers. Oral Oncol. 2019;97:76–81. 31437587 10.1016/j.oraloncology.2019.08.011

[9] Yu Y, Mabray M, Silveira W, Shen PY, Ryan WR, Uzelac A, et al. Earlier and more specific detection of persistent neck disease with diffusion-weighted MRI versus subsequent PET/CT after definitive chemoradiation for oropharyngeal squamous cell carcinoma. Head Neck. 2017;39(3):432–8. 27726241 10.1002/hed.24606

[10] Zhou S, Chan C, Rulach R, Dyab H, Hendry F, Maxfield C, et al. Long term survival in patients with human papillomavirus-positive oropharyngeal cancer and equivocal response on 12-week PET-CT is not compromised by the omission of neck dissection. Oral Oncol. 2022;128:105870. 35447564 10.1016/j.oraloncology.2022.105870

[11] Mathews F, Irizarry R, Rosenfeld R, Sundaram K. Systematic review and meta-analysis of post-treatment PET/CT in HPV-associated oropharyngeal cancer. Ann Otol Rhinol Laryngol. 2022;131(6):595–603. 34353135 10.1177/00034894211036842

[12] Ng SH, Liao CT, Lin CY, Chan SC, Lin YC, Yen TC, et al. Dynamic contrast-enhanced MRI, diffusion-weighted MRI and (18)F-FDG PET/CT for the prediction of survival in oropharyngeal or hypopharyngeal squamous cell carcinoma treated with chemoradiation. Eur Radiol. 2016;26(11):4162–72. 26911889 10.1007/s00330-016-4276-8

[13] Marzi S, Piludu F, Sanguineti G, Marucci L, Farneti A, Terrenato I, et al. The prediction of the treatment response of cervical nodes using intravoxel incoherent motion diffusion-weighted imaging. Eur J Radiol. 2017;92:93–102. 28624026 10.1016/j.ejrad.2017.05.002

[14] Wotman M, Ghaly M, Massaro L, Tham T, Seetharamu N, Kamdar D, et al. Improving post-CRT neck assessment in patients with HPV-associated OPSCC (review). Mol Clin Oncol. 2020;13(4):24. 32765872 10.3892/mco.2020.2094 PMC7403806

[15] van der Putten L, van den Broek GB, de Bree R, van den Brekel MW, Balm AJ, Hoebers FJ, et al. Effectiveness of salvage selective and modified radical neck dissection for regional pathologic lymphadenopathy after chemoradiation. Head Neck. 2009;31(5):593–603. 19132716 10.1002/hed.20987

[16] Thompson CA, Charlson ME, Schenkein E, Wells MT, Furman RR, Elstrom R, et al. Surveillance CT scans are a source of anxiety and fear of recurrence in long-term lymphoma survivors. Ann Oncol. 2010;21(11):2262–6. 20423914 10.1093/annonc/mdq215 PMC2962258

[17] Guo T, Qualliotine JR, Ha PK, Califano JA, Kim Y, Saunders JR, et al. Surgical salvage improves overall survival for patients with HPV-positive and HPV-negative recurrent locoregional and distant metastatic oropharyngeal cancer. Cancer. 2015;121(12):1977–84. 25782027 10.1002/cncr.29323 PMC4457566

[18] Lang Kuhs KA, Brenner JC, Holsinger FC, Rettig EM. Circulating tumor HPV DNA for surveillance of HPV-positive oropharyngeal squamous cell carcinoma: a narrative review. JAMA Oncol. 2023;9(12):1716–24. 37824111 10.1001/jamaoncol.2023.4042 PMC12011137

[19] Paolini F, Campo F, Iocca O, Manciocco V, De Virgilio A, De Pascale V, Moretto S, Dalfino G, Vidiri A, Blandino G, Pimpinelli F, Venuti A, Pellini R. It is time to improve the diagnostic workup of oropharyngeal cancer with circulating tumor HPV DNA: Systematic review and meta-analysis. Head Neck. 2023;45(11):2945–54. 10.1002/hed.27515. Epub 2023 Sep 16. 10.1002/hed.27515 37715656

[20] Akashi K, Sakai T, Fukuoka O, Saito Y, Yoshida M, Ando M, et al. Usefulness of circulating tumor DNA by targeting human papilloma virus-derived sequences as a biomarker in p16-positive oropharyngeal cancer. Sci Rep. 2022;12(1):572. 35022425 10.1038/s41598-021-04307-3 PMC8755847

[21] Berger BM, Hanna GJ, Posner MR, Genden EM, Lautersztain J, Naber SP, et al. Detection of occult recurrence using circulating tumor tissue modified viral HPV DNA among patients treated for HPV-driven oropharyngeal carcinoma. Clin Cancer Res. 2022;28(19):4292–301. 35576437 10.1158/1078-0432.CCR-22-0562 PMC9527497

[22] Chera BS, Kumar S, Shen C, Amdur R, Dagan R, Green R, Goldman E, Weiss J, Grilley-Olson J, Patel S, Zanation A, Hackman T, Blumberg J, Patel S, Thorp B, Weissler M, Yarbrough W, Sheets N, Mendenhall W, Tan XM, Gupta GP. Plasma Circulating Tumor HPV DNA for the Surveillance of Cancer Recurrence in HPV-Associated Oropharyngeal Cancer. J Clin Oncol. 2020;38(10):1050–8. 10.1200/JCO.19.02444. Epub 2020 Feb 4. Erratum in: J Clin Oncol. 2020 Oct 20;38(30):3579. 10.1200/JCO.20.02655. Erratum in: J Clin Oncol. 2023 Sep 20;41(27):4449. 10.1200/JCO.23.01228. 10.1200/JCO.19.02444 PMC7106982 32017652

[23] Ferrier ST, Tsering T, Sadeghi N, Zeitouni A, Burnier JV. Blood and saliva-derived ctDNA is a marker of residual disease after treatment and correlates with recurrence in human papillomavirus-associated head and neck cancer. Cancer Med. 2023;12(15):15777–87. 37526056 10.1002/cam4.6191 PMC10469655

[24] Haring CT, Bhambhani C, Brummel C, Jewell B, Bellile E, Heft Neal ME, Sandford E, Spengler RM, Bhangale A, Spector ME, McHugh J, Prince ME, Mierzwa M, Worden FP, Tewari M, Swiecicki PL, Brenner JC. Human papilloma virus circulating tumor DNA assay predicts treatment response in recurrent/metastatic head and neck squamous cell carcinoma. Oncotarget. 2021;12(13):1214–29. 10.18632/oncotarget.27992. 34194620 10.18632/oncotarget.27992 PMC8238244

[25] Jakobsen KK, Bendtsen SK, Pallisgaard N, Friborg J, Lelkaitis G, Gronhoj C, et al. Liquid biopsies with circulating plasma HPV-DNA measurements-a clinically applicable surveillance tool for patients with HPV-positive oropharyngeal cancer. Clin Cancer Res. 2023;29(19):3914–23. 37477909 10.1158/1078-0432.CCR-23-1064

[26] O'Boyle CJ, Siravegna G, Varmeh S, Queenan N, Michel A, Pang KCS, et al. Cell-free human papillomavirus DNA kinetics after surgery for human papillomavirus-associated oropharyngeal cancer. Cancer. 2022;128(11):2193–204. 10.1002/cncr.34109 PMC10032347 35139236

[27] Tatsumi M, Tanaka H, Takenaka Y, Suzuki M, Fukusumi T, Eguchi H, Watabe T, Kato H, Yachida S, Inohara H, Tomiyama N. Association of circulating tumor HPV16DNA levels and quantitative PET parameters in patients with HPV-positive head and neck squamous cell carcinoma. Sci Rep. 2024;14(1):3278. 10.1038/s41598-024-53894-4. 38332246 10.1038/s41598-024-53894-4 PMC10853198

[28] Warlow SJ, Adamowicz M, Thomson JP, Wescott RA, Robert C, Carey LM, Thain H, Cuschieri K, Li LQ, Conn B, Hay A, Nixon IJ, Aitman TJ. Longitudinal measurement of HPV copy number in cell-free DNA is associated with patient outcomes in HPV-positive oropharyngeal cancer. Eur J Surg Oncol. 2022;48(6):1224–34. 10.1016/j.ejso.2022.03.232. Epub 2022 Apr 6. 10.1016/j.ejso.2022.03.232 35431082

[29] Tanaka H, Takemoto N, Horie M, Takai E, Fukusumi T, Suzuki M, et al. Circulating tumor HPV DNA complements PET-CT in guiding management after radiotherapy in HPV-related squamous cell carcinoma of the head and neck. Int J Cancer. 2021;148(4):995–1005. 32895945 10.1002/ijc.33287

[30] Ferrandino RM, Chen S, Kappauf C, Barlow J, Gold BS, Berger MH, Westra WH, Teng MS, Khan MN, Posner MR, Misiukiewicz KJ, Bakst RL, Sindhu KK, Genden EM, Chai RL, Roof SA. Performance of Liquid Biopsy for Diagnosis and Surveillance of Human Papillomavirus-Associated Oropharyngeal Cancer. JAMA Otolaryngol Head Neck Surg. 2023;149(11):971–7. 10.1001/jamaoto.2023.1937. 37422913 10.1001/jamaoto.2023.1937 PMC10331620

[31] Hanna GJ, Roof SA, Jabalee J, Rettig EM, Ferrandino R, Chen S, et al. Negative predictive value of circulating tumor tissue modified viral (TTMV)-HPV DNA for HPV-driven oropharyngeal cancer surveillance. Clin Cancer Res. 2023;29(20):4306–13. 37566241 10.1158/1078-0432.CCR-23-1478 PMC10570676

[32] Li LQ, Adamowicz M, Wescott RA, Warlow SJ, Thomson JP, Robert C, et al. The role of liquid biopsy in management of the neck with indeterminate response on post-treatment imaging following non-surgical management of oropharyngeal cancer. Eur J Surg Oncol. 2023;49(1):55–9. 36244845 10.1016/j.ejso.2022.09.016

[33] Tanaka H, Suzuki M, Takemoto N, Fukusumi T, Eguchi H, Takai E, et al. Performance of oral HPV DNA, oral HPV mRNA and circulating tumor HPV DNA in the detection of HPV-related oropharyngeal cancer and cancer of unknown primary. Int J Cancer. 2022;150(1):174–86. 34486724 10.1002/ijc.33798 PMC9290341

[34] Creemers A, Ebbing EA, Pelgrim TC, Lagarde SM, van Etten-Jamaludin FS, van Berge Henegouwen MI, et al. A systematic review and meta-analysis of prognostic biomarkers in resectable esophageal adenocarcinomas. Sci Rep. 2018;8(1):13281. 30185893 10.1038/s41598-018-31548-6 PMC6125467

[35] Cargnin S, Canonico PL, Genazzani AA, Terrazzino S. Quantitative analysis of circulating cell-free DNA for correlation with lung cancer survival: a systematic review and meta-analysis. J Thorac Oncol. 2017;12(1):43–53. 27543256 10.1016/j.jtho.2016.08.002

[36] Campo F, Zocchi J, Moretto S, Mazzola F, Petruzzi G, Dona MG, et al. Cell-free human papillomavirus-DNA for monitoring treatment response of head and neck squamous cell carcinoma: systematic review and meta-analysis. Laryngoscope. 2021;132(3):560–8. 10.1002/lary.29739 34236084

[37] Deeks JJ, Altman DG. Diagnostic tests 4: likelihood ratios. BMJ. 2004;329(7458):168–9. 15258077 10.1136/bmj.329.7458.168 PMC478236

[38] Simundic AM. Measures of diagnostic accuracy: basic definitions. EJIFCC. 2009;19(4):203–11. 27683318 PMC4975285

[39] Glas AS, Lijmer JG, Prins MH, Bonsel GJ, Bossuyt PM. The diagnostic odds ratio: a single indicator of test performance. J Clin Epidemiol. 2003;56(11):1129–35. 14615004 10.1016/S0895-4356(03)00177-X

[40] Pellini R, Manciocco V, Turri-Zanoni M, Vidiri A, Sanguineti G, Marucci L, et al. Planned neck dissection after chemoradiotherapy in advanced oropharyngeal squamous cell cancer: the role of US, MRI and FDG-PET/TC scans to assess residual neck disease. J Craniomaxillofac Surg. 2014;42(8):1834–9. 25150165 10.1016/j.jcms.2014.06.023

[41] Harish K. Neck dissections: radical to conservative. World J Surg Oncol. 2005;3(1):21. 15836786 10.1186/1477-7819-3-21 PMC1097761

[42] Xie X, Ren Y, Wang K, Yi B. Molecular prognostic value of circulating Epstein-Barr viral DNA in nasopharyngeal carcinoma: a meta-analysis of 27,235 cases in the endemic area of Southeast Asia. Genet Test Mol Biomarkers. 2019;23(7):448–59. 31199710 10.1089/gtmb.2018.0304

[43] Rosenberg AJ, Izumchenko E, Pearson A, Gooi Z, Blair E, Karrison T, et al. Prospective study evaluating dynamic changes of cell-free HPV DNA in locoregional viral-associated oropharyngeal cancer treated with induction chemotherapy and response-adaptive treatment. BMC Cancer. 2022;22(1):17. 34980038 10.1186/s12885-021-09146-z PMC8722316

