# Supplementary material for: The landscape of circulating tumor HPV DNA and TTMV-HPVDNA for surveillance of HPV-oropharyngeal carcinoma: systematic review and meta-analysis
Source: J Exp Clin Cancer Res. 2024 Aug 3;43:215. doi: 10.1186/s13046-024-03137-1 (PMC11297591; doi:10.1186/s13046-024-03137-1)
Supplement: Supplementary file 1 — Supplementary Material 1: Table 1s. Algorithm for each database (MEDLINE, EMBASE, and Cochrane Library databases). [file 13046_2024_3137_MOESM1_ESM.docx]

| **Database** | **Research algorithm** | **N of articles** |
| --- | --- | --- |
| MEDLINE | (“TTMV” OR "circulating tumor HPV-DNA" OR "ctHPV-DNA" OR "cell free Human Papillomavirus-DNA" OR "cell free HPV-DNA" OR "cfDNA") AND ((("Head and Neck Neoplasms"[Mesh]) OR ("Head Neck Neoplasm*"[Title/Abstract] OR "Cancer* of Head and Neck"[Title/Abstract] OR "Head and Neck Cancer*"[Title/Abstract] OR "Cancer* of the Head and Neck"[Title/Abstract] OR "Head Neck Tumor*"[Title/Abstract])) OR (("Oropharyngeal Neoplasms"[Mesh]) OR ("Oropharyngeal Neoplasm*"[Title/Abstract] OR "Cancer* of Oropharnyx"[Title/Abstract] OR "Oropharyngeal* Cancer*"[Title/Abstract] OR "Cancer* of the Oropharynx"[Title/Abstract]))) | 121 |
| EMBASE | ('oropharynx tumor'/exp OR 'oropharyngeal neoplasms':ab,ti OR 'oropharyngeal tumor':ab,ti OR 'oropharyngeal tumour':ab,ti OR 'oropharynx tumour':ab,ti OR 'tumor, oropharyngeal':ab,ti OR 'tumour, oropharyngeal':ab,ti OR 'head and neck tumor'/exp OR 'ear nose throat tumor':ab,ti OR 'ear nose throat tumour':ab,ti OR 'ent tumor':ab,ti OR 'ent tumour':ab,ti OR 'head and neck neoplasms':ab,ti OR 'head and neck tumour':ab,ti OR 'head neck tumor':ab,ti OR 'head neck tumour':ab,ti OR 'orl tumor':ab,ti OR 'orl tumour':ab,ti OR 'otorhinolaryngeal tumor':ab,ti OR 'otorhinolaryngeal tumour':ab,ti OR 'otorhinolaryngologic neoplasms':ab,ti OR 'otorhinolaryngologic tumor':ab,ti OR 'otorhinolaryngologic tumour':ab,ti OR 'otorhinolaryngological tumor':ab,ti OR 'otorhinolaryngological tumour':ab,ti OR 'tumor, head and neck':ab,ti OR 'tumour, head and neck':ab,ti) AND ('ttmv':ab,ti OR 'ttmv' OR 'circulating tumor hpv-dna' OR 'cthpv-dna' OR 'cell free human papillomavirus-dna' OR 'cell free hpv-dna' OR 'cfdna') | 315 |
| Cochrane Library Databases | ("circulating tumor HPV-DNA" OR "ctHPV-DNA" OR "cell free Human Papillomavirus-DNA" OR "cell free HPV-DNA" OR "cfDNA") AND ((("Head and Neck Neoplasms"[Mesh]) OR ("Head Neck Neoplasm*"[Title/Abstract] OR "Cancer* of Head and Neck"[Title/Abstract] OR "Head and Neck Cancer*"[Title/Abstract] OR "Cancer* of the Head and Neck"[Title/Abstract] OR "Head Neck Tumor*"[Title/Abstract])) OR (("Oropharyngeal Neoplasms"[Mesh]) OR ("Oropharyngeal Neoplasm*"[Title/Abstract] OR "Cancer* of Oropharnyx"[Title/Abstract] OR "Oropharyngeal* Cancer*"[Title/Abstract] OR "Cancer* of the Oropharynx"[Title/Abstract]))) | 2 |

**Table 1s**: Algorithm for each database (MEDLINE, EMBASE, and Cochrane Library databases)
