# Supplementary material for: The landscape of circulating tumor HPV DNA and TTMV-HPVDNA for surveillance of HPV-oropharyngeal carcinoma: systematic review and meta-analysis
Source: J Exp Clin Cancer Res. 2024 Aug 3;43:215. doi: 10.1186/s13046-024-03137-1 (PMC11297591; doi:10.1186/s13046-024-03137-1)
Supplement: Supplementary file 2 — Supplementary Material 2: Table 2s. Assessment of methodological quality according to the Quality Assessment of Diagnostic Accuracy. [file 13046_2024_3137_MOESM2_ESM.docx]

Table 2s: Assessment of methodological quality according to the Quality Assessment of Diagnostic Accuracy

| Included studies | Risk of bias | | | | Applicability concerns | | |
| --- | --- | --- | --- | --- | --- | --- | --- |
|  | Patient  selection | Index  test | Reference  standard | Flow and  timing | Patient  selection | Index  test | Reference  standard |
| Jakobsen, K., K. et al (2023) | + | ? | + | ? | + | - | - |
| Ferrier, S., T. et al (2023) | + | ? | + | ? | + | - | - |
| Akashi et al (2022) | + | ? | + | + | + | - | - |
| Tanaka et al (2023) | + | ? | + | + | + | - | - |
| Warlow et al (2022) | + | ? | + | ? | + | - | - |
| Berger et al (2020) | + | ? | + | + | + | - | - |
| Ferrandino et al (2023) | - | ? | - | ? | - | - | - |
| Chera et al /2020 | + | ? | + | ? | + | - | - |
| Haring et al (2021) | + | ? | + | ? | + | - | - |
| O’ Boyle et al (2022) | + | ? | + | ? | + | - | - |
| Tanaka et al (2021) | + | ? | + | + | + | - | - |
| Hanna et al (2023) | - | ? | + | + | - | - | - |

Key: +:low risk; ?: unclear risk; -: high risk.
